# Pubertal maturation and sex effects on the default-mode network connectivity implicated in mood dysregulation

**DOI:** 10.1038/s41398-019-0433-6

**Published:** 2019-02-25

**Authors:** Monique Ernst, Brenda Benson, Eric Artiges, Adam X. Gorka, Herve Lemaitre, Tiffany Lago, Ruben Miranda, Tobias Banaschewski, Arun L. W. Bokde, Uli Bromberg, Rüdiger Brühl, Christian Büchel, Anna Cattrell, Patricia Conrod, Sylvane Desrivières, Tahmine Fadai, Herta Flor, Antoine Grigis, Juergen Gallinat, Hugh Garavan, Penny Gowland, Yvonne Grimmer, Andreas Heinz, Viola Kappel, Frauke Nees, Dimitri Papadopoulos-Orfanos, Jani Penttilä, Luise Poustka, Michael N. Smolka, Argyris Stringaris, Maren Struve, Betteke M. van Noort, Henrik Walter, Robert Whelan, Gunter Schumann, Christian Grillon, Marie-Laure Paillère Martinot, Jean-Luc Martinot, for the IMAGEN Consortium, J Dalley, N Subramaniam, D Theobald, C Bach, G. J Barker, M Fauth-Bühler, S Millenet, R Spanagel, L Albrecht, N Ivanov, M Rapp, J Reuter, N Strache, A Ströhle, J. B Poline, Y Schwartz, B Thyreau, J Ireland, J Rogers, N Bordas, Z Bricaud, I Filippi, A Galinowski, F Gollier-Briant, D Hall, S Havatzias, T Jia, C Mallik, C Nymberg, B Ruggeri, L Smith, K Stueber, L Topper, H Werts, R Brühl R, A Ihlenfeld, B Walaszek, T Hübner, K Müller, T Paus, S Ripke, E Mennigen, D Schmidt, N. C Vetter, V Ziesch, D Carter, C Connolly, S Nugent, J Jones, J Yacubian, S Schneider, K Head, N Heym, C Newman, Z Pausova, A Tahmasebi, D Stephens

**Affiliations:** 1 NIMH/NIH, Bethesda, MD USA; 2 INSERM, UMR 1000, Research unit “Neuroimaging and Psychiatry”, DIGITEO Labs, University Paris-Saclay, and University Paris Descartes, Gif sur Yvette, France; 3 INSERM, UMR 1000, Faculté de médecine, University Paris-Saclay, DIGITEO Labs, Gif sur Yvette, France; 4 University Paris Descartes, Paris, France; 5 Center for Neuroimaging Research (CENIR), Brain & Spine Institute, Paris, France; 6 Psychiatry Department 91G16, Orsay Hospital, Paris, France; 7 Department of Child and Adolescent Psychiatry and Psychotherapy, Central Institute of Mental Health, Medical Faculty Mannheim, Heidelberg University, Mannheim, Germany; 8 Discipline of Psychiatry, School of Medicine and Trinity College Institute of Neurosciences, Trinity College, Dublin, Ireland; 9 University Medical Centre Hamburg-Eppendorf, House W34, 3.OG, Hamburg, Germany; 10 Physikalisch-Technische Bundesanstalt, Abbestr. 2 - 12, Berlin, Germany; 11 Medical Research Council - Social, Genetic and Developmental Psychiatry Centre, Institute of Psychiatry, Psychology & Neuroscience, King’s College London, London, United Kingdom; 12 Department of Psychological Medicine and Psychiatry, Institute of Psychiatry, Psychology & Neuroscience, King’s College London, London, United Kingdom; 13 Department of Psychiatry, Université de Montréal, CHU Ste Justine Hospital, Montréal, QC Canada; 14 Department of Psychology, School of Social Sciences, University of Mannheim, 68131 Mannheim, Germany; 15 Neurospin, Commissariat à l’Energie Atomique, CEA-Saclay Center, Saclay, France; 16 Department of Psychiatry and Psychotherapy, University Medical Center Hamburg-Eppendorf, Martinistr. 52, 20246 Hamburg, Germany; 17 Departments of Psychiatry and Psychology, University of Vermont, 05405 Burlington, VT USA; 18 Sir Peter Mansfield Imaging Centre School of Physics and Astronomy, University of Nottingham, University Park, Nottingham, United Kingdom; 19 Department of Psychiatry and Psychotherapy, Campus CharitéMitte, Charité-Universitätsmedizin Berlin, Charitéplatz 1, Berlin, Germany; 20 Department of Child and Adolescent Psychiatry Psychosomatics and Psychotherapy, Campus CharitéMitte, Charité-Universitätsmedizin Berlin, Charitéplatz 1, Berlin, Germany; 21 Department of Social and Health Care, Psychosocial Services Adolescent Outpatient Clinic, University of Tampere, Kauppakatu 14, Lahti, Finland; 22 Department of Child and Adolescent Psychiatry and Psychotherapy, Medical University of Vienna, Vienna, Austria; 23 Department of Psychiatry and Neuroimaging Center, Technische Universität Dresden, Dresden, Germany; 24 Department of Child and Adolescent Psychiatry, Institute of Psychiatry, Psychology & Neuroscience, King’s College London, London, United Kingdom; 25 Department of Psychology, University College, Dublin, Ireland; 26 AP-HP, Department of Child and Adolescent Psychiatry, Pitié-Salpêtrière Hospital, Paris, France; 27 Sorbonne Universités, Paris, France

## Abstract

This study examines the effects of puberty and sex on the intrinsic functional connectivity (iFC) of brain networks, with a focus on the default-mode network (DMN). Consistently implicated in depressive disorders, the DMN’s function may interact with puberty and sex in the development of these disorders, whose onsets peak in adolescence, and which show strong sex disproportionality (females > males). The main question concerns how the DMN evolves with puberty as a function of sex. These effects are expected to involve within- and between-network iFC, particularly, the salience and the central-executive networks, consistent with the Triple-Network Model. Resting-state scans of an adolescent community sample (*n* = 304, male/female: 157/147; mean/std age: 14.6/0.41 years), from the IMAGEN database, were analyzed using the AFNI software suite and a data reduction strategy for the effects of puberty and sex. Three midline regions (medial prefrontal, pregenual anterior cingulate, and posterior cingulate), within the DMN and consistently implicated in mood disorders, were selected as seeds. Within- and between-network clusters of the DMN iFC changed with pubertal maturation differently in boys and girls (puberty-X-sex). Specifically, pubertal maturation predicted weaker iFC in girls and stronger iFC in boys. Finally, iFC was stronger in boys than girls independently of puberty. Brain–behavior associations indicated that lower connectivity of the anterior cingulate seed predicted higher internalizing symptoms at 2-year follow-up. In conclusion, weaker iFC of the anterior DMN may signal disconnections among circuits supporting mood regulation, conferring risk for internalizing disorders.

## Introduction

Puberty and sex critically influence brain maturation in adolescence (for review, see ref. ^1,2^). The pubertal rise in sex steroids is thought to further refine the organizational sex differences that are established early in life (for review, see ref. ^3,4^. These puberty-related effects are expected to contribute to brain development and to promote sex differences in neural circuits. Consistent with this notion, puberty-related changes in brain functional organization would be predicted to reveal different trajectory patterns between girls and boys. The influence of puberty on brain function may also have a role in the emergence of psychiatric disorders, for two reasons. First, incidence rates of psychiatric disorders peak in adolescence^5^, and, second, they exhibit striking sex differences^6^, such as female preponderance for depressive and anxiety disorders. In addition, a specific role of sex steroids, such as high levels of dehydroepi–androsterone in children, has been associated with mental health problems (e.g., ref. ^7^).

In contrast to the abundant neurodevelopmental research probing neural changes with age and sex (e.g., ref. ^8–18^), neuroimaging studies that query the effects of puberty on neural development, are relatively sparse^19–26^ (for review, see ref. ^27^). A primary focus of these neurodevelopmental studies have targeted structural measures of the brain (e.g., ref. ^28–31^ and task-based functional magnetic resonance imaging (fMRI)^20–24^). Relatively few resting-state fMRI studies have investigated the effects of age and sex in adolescence. Across the relative sparsity of these studies, many different methods and targets have been used, which make the identification of consistent patterns too soon to draw. Some studies focus on specific structures. For example, Alarcon et al.^23^ examined iFC of subregions of the amygdala (seeds) in 122 healthy youths (10–16 years). Their findings show opposite age-related changes in connectivity in girls vs. boys, but these changes are not always in the same direction depending on the amygdala subregions. Others have conducted studies of whole-brain organization, using methods such as graph theory (for review ref. ^32^). For example, Satterthwaite et al. examined sex differences in 9–22-year-old youths^33^. They reported sex differences in the organization of whole-brain connectivity (i.e., greater between-networks connectivity in boys, and greater within-networks connectivity in girls). This type of data underscore the presence of sex differences in brain connectivity in youths, but does not speak specifically to the direction of sex differences in specific networks. Unfortunately, an analogous situation characterizes this type of research in adults (e.g., ^34–36^). Of note, a recent study using a large adult sample from the human connectome (*n* = 820, 336 females, 22–37 yo) identified the default-mode network (DMN) as being the best predictor of sex status, particularly for couplings involving the fusiform gyrus and ventromedial prefrontal cortex^37^. The direction of effects was not detailed. Taken together, this brief survey of the literature does not permit to integrate existing findings into specific hypotheses that could guide the present work. Finally, to our knowledge, no studies have yet investigated puberty-related changes in resting-state functional connectivity (referred to as “intrinsic Functional Connectivity” or iFC), particularly with respect to the DMN, which is shown to have a central role in the development of psychopathology^38^, and seems to be highly sensitive to sex status^37^. Of note, one reason for the sparsity of research on puberty stems from the difficulty of dissociating the effects of age from puberty.

The present study takes advantage of a large community cohort of adolescents, all ~14-year old^39^, to examine how puberty and sex influence the iFC of three specific nodes. These three nodes have been selected for two reasons. First, their structural parameters have been associated with adolescent mood dysregulation^40,41^. Second, they belong to the DMN^42,43^. As mentioned above, the DMN has been consistently implicated in internalizing disorders (e.g., ref. ^44–48^ and it comprises midline cortical regions that systematically emerge in studies of mood disorders (see reviews and meta-analyses^45,49–54^). Therefore, understanding the effects of puberty and sex on the development of the DMN function might shed light on the neural mechanisms conferring vulnerability to mood dysregulation in adolescence.

Highly relevant to this question is the Triple-Network Model^55^. This model proposes that dysfunction or imbalance among three core canonical networks of resting-state fMRI might contribute to a number of psychiatric disorders. These networks consist of the DMN, the central-executive network and the salience network (SN). The DMN serves self-referential-related functions and comprises regions in the anterior medial prefrontal cortex (PFC), posterior cingulate cortex, middle temporal cortex, and hippocampus^47,48^. The central-executive network supports working memory, decision-making, and cognitive control. This latter network is particularly important for the regulation of emotion processing, which itself depends largely on subcortical regions (e.g., amygdala). The central-executive network encompasses the dorsolateral PFC and dorsomedial PFC^56–58^. Finally, the SN supports the integration of internal and external stimuli into emotional and behavioral responses. The core nodes of the SN include the insula and dorsal anterior cingulate cortex^59^. The framework of the Triple-Network Model is used as a heuristic tool in the present work. This approach emulates the widely use of neural systems models to explain typical adolescent behaviors, such as increased risk-taking, emotional lability, or social transformation^60–64^.

The present work focuses on how puberty and sex affect the DMN iFC, including couplings within and between networks, especially the salience and central-executive networks of the Triple-Network Model. We hypothesize sex differences in the pubertal maturation effects on the brain’s iFC. We also anticipate sex differences that are independent of pubertal maturation^1^. However, we do not predict pubertal maturation to influence brain connectivity similarly in males and females, because puberty is by essence sexually dimorphic. Specific directional hypotheses are difficult to predict, based on the lack of prior work of typical changes of resting-state networks with puberty. Finally, exploiting the 2-year behavioral follow-up (16 yo) of this cohort, exploratory brain–behavior analyses are expected to reveal associations between the DMN iFC of the 14-year old and behavioral dimensions of internalizing, externalizing, or social problems when these adolescents reach 16 years of age.

## Participants and methods

### Participants

The IMAGEN consortium recruited over 2000 youths. Only five sites opted to collect resting-state scans. IMAGEN data are available from a dedicated database: https://imagen2.cea.fr. Adolescents (*n* = 381) from the IMAGEN sample^39^ underwent fMRI scanning in a resting state. A sample size of *n* = 381 subjects was expected to provide sufficient power to detect reliable interaction effects of puberty by sex on iFC measures. Indeed, previous studies have reported significant clinical group effects on measures of intrinsic connectivity in pediatric samples smaller than *n* = 60 (e.g., ref. ^65–67^). Recruitment and assessment procedures, and exclusion and inclusion criteria are described elsewhere in detail^39^. In brief, participants and their parents were recruited via middle-schools in five European sites. Inclusion criterion was age between 13 and 15 years. Exclusion criteria were birth weight < 800 g, severe medical conditions, bipolar disorder, treatment for schizophrenia, and major neuro-developmental disorders. All participants were assessed for intelligence quotient (IQ) using the Wechsler Abbreviated Scale of Intelligence^68^.

Written informed assent and consent were obtained, respectively, from all adolescents and their parents in accordance with the ethics committees of the participating institutions^39^ and the Declaration of Helsinki. Seventy-seven participants were excluded from the analysis owing to excessive head motion (i.e., > 30% of acquired Repetition Time (TRs) with a frame-to-frame Euclidean norm motion derivative > 0.25 mm; *n* = 72, 53 boys and 19 girls), poor spatial normalization by visual inspection (*n* = 2), or corrupted data (*n* = 1). Two participants lacked pubertal scores. The excluded group (*n* = 77), compared to included participants (*n* = 304), had more boys (*T* = 3.58, *p* < 0.001), but was similar in age, puberty status, and IQ (all *p*’s > 0.1). Characteristics of the sample are presented in Table 1. Of note, participants overlapped slightly with those of our previous structural studies (i.e., *n* = 21 in common with^40^, *n* = 31 in common with Vulser et al.^41^, and *n* = 5 in common to all three samples).

**Table 1:** Demographic information

	All participants		Male participants		Female participants	
Demographics no. of participants	*N* = 304		*N* = 157	(51.6%)	*N* = 147	(48.4%)
	Mean	(±1 SD)	Mean	(±1 SD)	Mean	(±1 SD)
Age (days)	5279	± 151	5265	± 149	5294	± 152
Puberty (PDS)^a^	2.86	± 0.57	2.56	± 0.55	3.18	± 0.38
IQ^b^	107	± 12	107	± 12	108	± 12
Scanner site 1 (Dublin)	*N* = 26		*N* = 8		*N* = 18	
Scanner site 2 (London)	*N* = 42		*N* = 42		*N* = 0	
Scanner site 3 (Dresden)	*N* = 116		*N* = 57		*N* = 59	
Scanner site 4 (Mannheim)	*N* = 54		*N* = 19		*N* = 35	
Scanner site 5 (Paris)	*N* = 66		*N* = 31		*N* = 35	

*PDS* Pubertal Development Scale, IQ intelligent quotient. Demographic information for the whole sample, male participants, and female participants

^a^(females > males, *p* < 0.05)

^b^IQ measured using the Wechsler Abbreviated Scale of Intelligence (WASI)

Behavioral data were collected again in this sample 2 years later, at age 16 years. The attrition rate was 17% (53 subjects were not tested at follow-up), leaving a sample of 251 16-yo adolescents.

### Behavioral assessments

Every participant completed a psychiatric assessment via the Development and Well-Being Assessment (DAWBA, www.dawba.com). The DAWBA is a self-administered questionnaire consisting of open- and close-ended questions completed by the participants and their parents that generates computerized probability levels of meeting DSM-IV and ICD-10 diagnoses, called “DAWBA bands” that are subsequently validated by experienced clinicians^69^. As part of the DAWBA, participants also completed a self-report inventory behavioral screening questionnaire, the Strengths and Difficulties questionnaire (SDQ^70^), which gives a measure of severity of problems within internalizing, externalizing, and social behavioral domains. Although the resting-state fMRI scanning only occurred at age 14 years, the behavioral assessments were repeated 2 years later, at age 16 years.

### Pubertal status

Pubertal status was assessed via self-report using the Pubertal Development Scale (PDS)^71^. Previous research has demonstrated that the PDS exhibits good internal consistency (median *α* = 0.77) and is highly correlated with physician ratings (Pearson’s *R* = 0.61)^71,72^.

### BOLD fMRI data acquisition, preprocessing, and analysis

MRI data were acquired at five sites using 3 T scanners: Phillips (Dublin), General Electrics (London), and Siemens (Paris, Dresden, Mannheim). BOLD fMRI signal was acquired across 40 interleaved slices using the following parameters: TR = 2,200 ms; TE = 30 ms; flip angle = 75°; acquisition matrix = 64 × 64 × 40 with 2.4 mm slice thickness and 1 mm slice gap yielding an acquisition resolution 3.4 mm isotropic; 187 volumes collected over 6.5 minutes. High-resolution anatomical images were obtained using parameters based on the ADNI protocol, yielding a final voxel size of 1.1 × 1.1 × 1.1 mm.

Preprocessing and analyses of BOLD fMRI data were conducted using AFNI^73^. FreeSurfer version 5.3^74^ was employed to segment the T1-weighted anatomical images. The ^i^first four volumes of the functional run were discarded to allow for steady-state equilibrium. Functional volumes were slice-time corrected, aligned, and co-registered to the participants’ corresponding anatomical image. Functional volumes were then normalized to the Colin 27 Average Brain standardized template using 3dQwarp, which is a nonlinear transformation, and spatially smoothed with a 6 mm Full-Width Half-Maximum (FWHM) Gaussian kernel. All coordinates are reported in the Talairach and Tournoux system^75^.

Acknowledging the debate as to whether global signal should be regressed out of resting-state data sets, we decided not to adopt this strategy. This decision was based on Saad et al.^76^ who suggest that global signal regression can introduce spurious correlations into resting-state data sets, and advise against its use in preprocessing. In addition, we employed several strategies to minimize motion- and physiological-related variance, thus mitigating the need to apply additional measures, like global signal regression, to minimize variance related to motion and cardiac/respiratory processes (Power et al.^77^).

The following nuisance signals were regressed from the functional volumes: (1) six head motion parameters and their derivatives, (2) average time-series extracted from the ventricles, (3) time-series from local white matter within a 25-mm radius sphere surrounding each voxel using the ANATICOR approach^78^, and (4) individual regressors corresponding with a frame-to-frame Euclidean norm motion derivative ≥ 0.25 mm, or volumes where ≥ 10% of voxels were determined to be outliers. This strategy followed the recommendations of Power et al.^79^.

Three cortical regions of interest (ROIs) from the DMN^80^ were selected as seeds. These three seeds were retained because of their previously demonstrated association with subthreshold elevated^40^, as well as depressed^41^ mood symptoms in two independent subsets of the community cohort of 14 yo from the IMAGEN consortium (https://imagen.cea.fr^39^). MNI coordinates were all converted to Talairach coordinates using the Yale mni2tal GUI (“MNI- Yale University” 2017). Specifically, cubic ROIs (3 mm × 3 mm × 3 mm) were created within the left pregenual ACC (lpgACC; Talairach: *x* = −12, *y* = 36, *z* = 12), left medial PFC (lmPFC; Talairach: *x* = −2, *y* = 45, *z* = 16), and the left PCC (Talairach: *x* = −1, *y* = −47, *z* = 34^81^). The left side was selected because the regions identified in previous studies were on the left side. Generically, these cortical regions have been implicated in the processing of salience and emotion encoding, particularly in the context of social and self-referential information (mPFC)^82^, visual stimuli (PCC)^83^, and autonomic visceral signals (pgACC)^84^.

Subsequently, average time-series from seed ROIs were extracted from the residualized functional images and were used to calculate Pearson correlations between the time-series from the seed ROIs and every voxel in the brain. Resulting statistical images were Fisher-transformed for group analyses.

ANCOVA models (3dMVM) were used to determine the interaction of puberty-X-sex and the main effects of each factor^85^. All group-level ANCOVA analyses statistically controlled for the effects of scanner site (Dublin, Dresden, London, Mannheim, and Paris), as well as age. Group-level analyses were limited to all gray matter regions as determined by the parcellation of the Colin 27 Average Brain (i.e., CA_N27_ML atlas). Statistical thresholding was calculated using 3dClustSim’s Monte Carlo simulation via updated versions of 3dFWHMx and 3dClustSim to address the concerns of inflated false positive rates identified by Eklund^86^. These updates incorporate a mixed autocorrelation function that better models non-Gaussian noise structure^87^. The resulting maps were thresholded to *p* < 0.005 two-tailed, *k* = 37, which represents a global cluster correction at *p* < 0.05. Finally, to examine more stringently potential effects of group motion, we conducted an additional analysis including the additional covariate of individual average motion per TR. This analysis is presented in supplemental material (Table S1).

Average iFC values from clusters exhibiting a puberty-X-sex interaction, or main effects of sex or puberty, were extracted using 3dmaskave. *Post hoc* comparisons were conducted within SPSS v24 and interactions were probed using the PROCESS v216^88^ macro by calculating the beta value for the relationship between puberty and iFC for male and female participants, while controlling for age and scanner site. Levene’s test for equality of variances, and measures of skew and kurtosis are reported in Supplemental Table S2. Importantly, all clusters exhibiting an interaction between puberty and sex are normally distributed. Furthermore, although variance is not equal between groups for all identified clusters, multiple regression analyses, which do not entail equality of variance assumptions, demonstrate that identified interactions remain significant within a multiple regression framework (Supplemental Tables S3–S4).

### Brain–behavior analyses based on factorial approach

To reduce the number of variables, two sets of principal components using SAS-9.4 program were extracted from the neural data at baseline (14 years old), and the behavioral data collected at 2-year follow-up (16 years old). All the iFC clusters significantly modulated by puberty and/or sex were entered into one factor analysis, and 15 items of the SDQ into a separate factor analysis. These factor analyses used ones as prior communality estimates. The principal axis method was employed to extract the components, and this was followed by a varimax (orthogonal) rotation.

Only two DAWBA bands were retained for this analysis, generalized anxiety disorder and depression, based on our main interest in these frequently comorbid diagnoses^89,90^. The SDQ has 33 items. A total of 16 items were removed a priori, because of no interest. These no-interest items included nine positive items (e.g., “considerate”), seven general items (e.g., “impact at home”). Two additional items (“clingy”, “unhappy”) were removed because they loaded on multiple factors. Accordingly, the final factor analysis was conducted on 15 items of the SDQ and the two DAWBA bands that probe generalized anxiety disorder and depression. These items and corresponding factor loadings are presented in Table 2. A three-factor resolution was found to be optimal. Combined, factors 1, 2, and 3 accounted for 30%, 29%, and 21% of the total variance respectively. When interpreting the rotated factor pattern, an item was said to load on a given factor if the factor loading was 0.40 or greater for that factor and < 0.40 for the others. Using these criteria, six items were found to load on the first factor, which was subsequently labeled “internalizing symptoms”. Five items were found to load on the second factor, which was subsequently labeled “externalizing symptoms”. Finally, three items were found to load on the third factor, which was subsequently labeled “social problems”.

**Table 2:** Principal component analysis of behavioral variables from the strengths and difficulties questionnaire (SDQ^59^), and the development and well-being assessment (DAWBA, www.dawba.com*)* at follow-up (16 yo) and variance explained by each factor

Variance explained by each factor		
Internalizing	externalizing	Social problems
2.9905752	2.9182511	2.1157313

	Factor-1	Factor-2	Factor-3
	Internalizing	Externalizing	Social
Somatic	52^a^	22	10
Worries	63^a^	8	16
Afraid	67^a^	13	9
Impact	72^a^	20	14
Depression^b^	69^a^	18	−14
Generalized anxiety ^b^	81^a^	13	8
Fidgety	16	75^a^	−2
Restless	15	78^a^	−1
Distractible	13	67^a^	−10
Conduct problems	24	50^a^	16
Hyperactive	19	94^a^	−6
Solitary	−4	−3	79^a^
Relates better to adults	17	−3	70^a^
Peer problems	20	2	93^a^

Printed values are multiplied by 100 and rounded to the nearest integer. Values > 0.4 are flagged by an ‘a’

The SDQ gives a measure of severity of problems within internalizing, externalizing, and social behavioral domains

^b^DAWBA band: the DAWBA bands represent the probability levels of meeting DSM-IV and ICD-10 diagnoses

A similar approach was adopted to reduce the number of neural variables (iFC clusters). As reported below in the results, 17 clusters were of interest. Four clusters were removed because they loaded on more than one factor. The final 13 clusters and corresponding factor loadings are presented in Table 3. The optimal solution was a three-factor model. Factors 1, 2, and 3 accounted for 49%, 30%, and 27% of the total variance, respectively. The interpretation of the rotated factor pattern, used the same threshold as above, i.e., 0.40. Using these criteria, eight clusters were found to load on the first factor. These eight clusters were iFC clusters of the lPCC seed (*n* = 5 clusters) and of the lMPFC seed (*n* = 3 cluster), all of which being modulated by Puberty-X-Sex. Therefore, factor-1 was labeled PCC/mPFC-pubXsex. Four clusters loaded on the second factor. All these clusters belonged to the lPCC-seed iFC, and were sensitive to Sex. Factor-2 was labeled PCC-sex Four clusters loaded on the third factor that was represented by clusters of the lACC seed. All these clusters were modulated by Sex. Factor-3 was thus labeled ACC-Sex.

**Table 3:** Principal component analysis of the intrinsic functional connectivity of the three seeds influenced by sex and puberty-X sex(14 yo), and variance explained by each factor

Variance explained by each factor		
PCC/mPFC-pubXsex	PCC-sex	ACC-sex
4.8571200	2.9548272	2.7282190

	Rotated factor pattern		
	Factor-1	Factor-2	Factor-3
	PCC/mPFC-pubXsex	PCC-sex	ACC-sex
lpgACC-seed sex			
L_dlPFC	28	31	69^a^
L_dmPFC	12	7	81^a^
L-thalamus	15	5	65^a^
R_PCC	9	21	77^a^
lPCC-seed sex			
L_insula	22	84^a^	23
R_mTemporal	39	55^a^	31
R_sTemporal	33	72^a^	8
R_insula	35	85^a^	15
lmPFC/lPCC-seed pubertyXsex			
L_dmPFC	67^a^	30	39
L_Temporal	71^a^	19	10
R_dlPFC	59^a^	26	30
L_infParietal	77^a^	16	5
L_precen	76^a^	34	16
R_mTemporal	81^a^	25	9
R_dmPFC	78^a^	17	28
R_precen	76^a^	38	17

Printed values are multiplied by 100 and rounded to the nearest integer. Values > 0.4 are flagged by an ‘a’

Labels: l = left, r = right

pgACC: pregenual anterior cingulate cortex; dLPFC: dorsolateral prefrontal cortex; dmPFC: dorsomedial; PCC: posterior cingulate cortex; mPFC: medial prefrontal cortex; mTemporal: middle temporal cortex; sTemporal: superior temporal cortex; rdmPFC: right dorsomedial prefrontal cortex; precen: precentral cortex

Three multiple regression analyses were conducted to assess associations between the baseline iFC neural factors and each of the three follow-up (16 yo) behavioral factors.

## Results

### Puberty-X-sex interactions

**Fig. 1:**
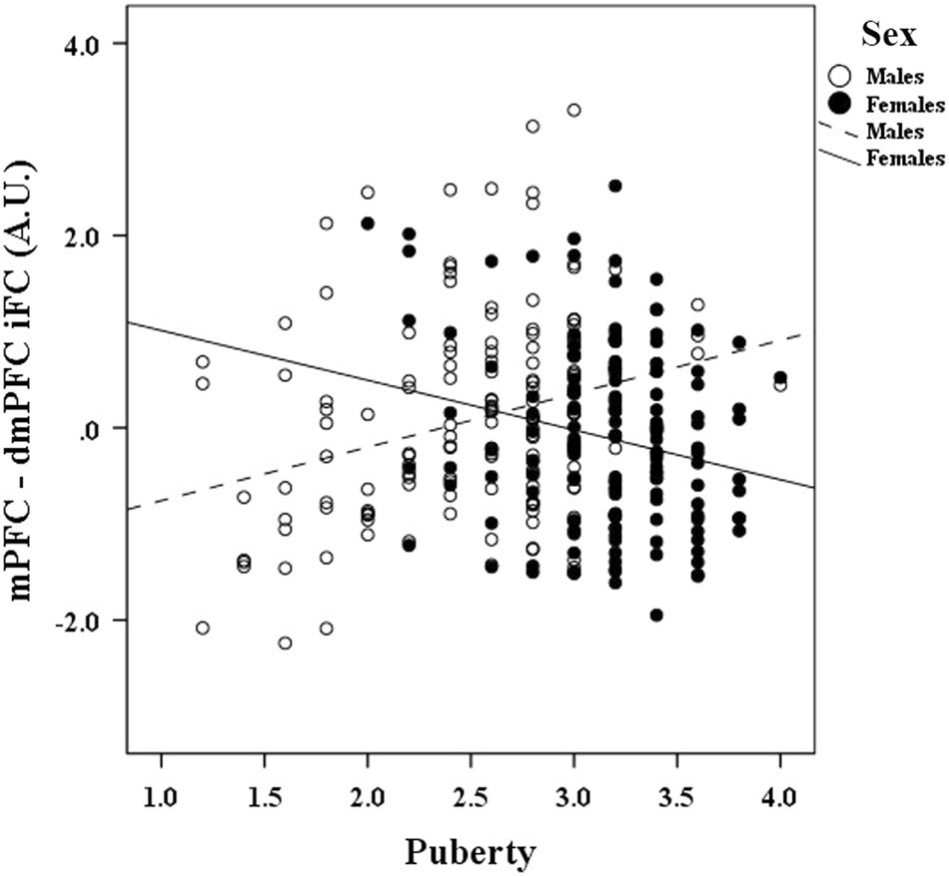
Scatterplot illustrating a representative relationship between puberty and the iFC of the central-executive network (central-executive network), as a function of sex Specifically, pubertal development (*x* axis) is positively associated with the mPFC iFC with dmPFC (*y* axis) in boys, but negatively associated with the iFC between these regions in girls

All significant puberty-X-sex interactions indicated that pubertal maturation was associated with increasingly weaker connectivity in females, but increasingly stronger connectivity in males. Slopes between puberty and iFC are reported separately for boys and girls in Table 4, and a representative pattern is illustrated in the scatterplot of Fig. 1.

**Table 4:** Decomposition of the Puberty by Sex significant effects on iFC

Effect of Puberty on iFC	Boys		Girls	
**lmPFC seed**	***β*** **value**	***P*** **value**	***β*** **value**	***P*** **value**
dmPFC	0.080	<0.0005	−0.073	<0.05
Middle temporal gyrus	0.062	<0.005	−0.084	<0.01
Dorsolateral PFC	0.038	=0.071	−0.100	<0.005
**lPCC seed**				
Inferior parietal lobule	0.074	<0.005	−0.104	<0.005
Left precentral gyrus	0.046	<0.05	−0.108	<0.001
Right Precentral gyrus	0.040	=0.089	−0.114	<0.001
dmPFC	0.075	<0.005	−0.078	<0.05
Middle temporal gyrus	0.073	<0.005	−0.103	<0.01

Correlations between puberty and significant iFC clusters are shown separately for boys and girls, for the lmPFC seed and the lPCC seed. Beta coefficients and *p* values associated with the simple slopes between pubertal development and iFC are presented separately for boys (middle column) and girls (right column). All models presented in this table control for age and scanner site

mPFC: medial prefrontal cortex; dmPFC: dorsomedial prefrontal cortex; PCC: posterior cingulate cortex

**Fig. 2:**
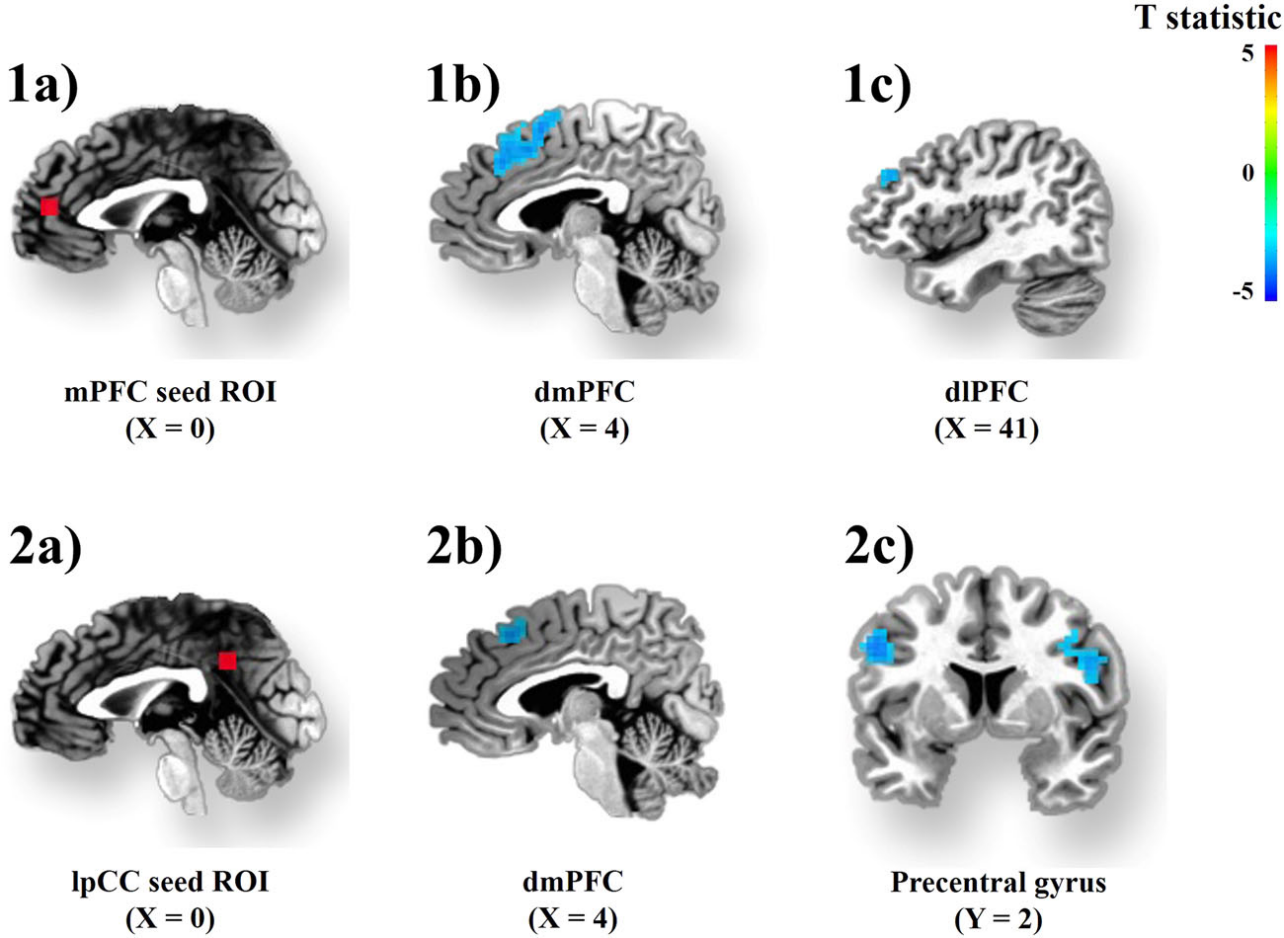
Pubertal development is associated with the iFC of the central-executive network (central-executive network), as a function of sex Upper panel: interactions between puberty and gender characterizing the iFC between the mPFC seed ROI (1**a**), clusters within the dmPFC (1**b**), and dlPFC (1**c**). Not depicted, is the right middle temporal cortex. Bottom panel: interactions between puberty and sex characterizing the iFC between the pCC seed ROI (2**a**), the dmPFC (2**b**), and the bilateral precentral gyrus (2**c**). Not depicted are the right middle temporal and left inferior parietal cortex

The anterior (lmPFC) and posterior (lPCC) midline cortical seeds revealed a similar pattern of clusters affected by the puberty-X-sex interaction (Table 5). These clusters were within the DMN and between the default-mode and central-executive networks. The within-DMN clusters included regions in the middle temporal gyrus (BA37) for both seeds, and the inferior parietal lobule (BA40) for the lPCC seed only. The between networks (default-mode with central-executive) clusters were found in the precentral gyrus for both seeds, the frontal eye field (FEF, BA 8) for the lPCC seed, and the dorsolateral PFC (BA 9) for the lmPFC seed (Fig. 2).

**Table 5:** Significant iFC of three seeds, left medial PFC, left anterior cingulate cortex, and left posterior cingulate cortex across the whole brain

					Talaraich		
		Region	Cluster Size (k)	Maxima	X	Y	Z
		Puberty by sex					
lMPFC seed	DMN–CEN	L dmPFC (BA 6)	223	17.16	−4.5	4.5	56.5
		R dlPFC (BA 9)	36	11.87	40.5	34.5	29.5
	DMN–DMN	R mTemporal ctx	41	16.10	58.5	−56	−6.5
lPCC seed	DMN–CEN	R dmPFC (BA 8)	47	16.28	7.5	25.5	44.5
		L Precentral Ctx (BA 6)	61	15.30	−47	1.5	32.5
		R Precentral Ctx (BA 6)	70	12.28	46.5	1.5	26.5
	DMN–DMN	R mTemporal Ctx	68	16.42	58.5	−53	−9.5
		L Inferior Parietal Ctx	82	24.38	−44	−47	41.5
		Sex					
lMPFC seed		R Occipital Cx	37	17.97	31.5	−89	17.5
lPCC seed	DMN–SN	L Insula	41	22.34	−38	−23	11.5
		R Insula	89	16.55	46.5	−14	11.5
	DMN–DMN	R mTemporal Ctx	56	13.37	43.5	−59	14.5
		R aTemporal Ctx	47	16.42	58.5	7.5	−6.5
lpgACC seed	DMN–CEN	L mPFC (BA 10)	142	16.60	−1.5	55.5	14.5
		L dlPFC (BA 9)	102	15.57	−50	16.5	26.5
	DMN–DMN	R PCC	37	15.35	13.5	−68	14.5
		L Thalamus	48	17.79	−7.5	−23	8.5

DMN–DMN reflects within-network iFC, whereas DMN–CEN, and DMN–SN reflect between-network iFC

Of note, the occipital cortex and the thalamus are not associated with specific resting-state networks

The lpgACC-iFC showed no significant clusters in puberty-X-sex analyses.

### Sex main effect

All sex effects followed the same pattern, i.e., stronger iFC in boys than girls.

**Fig. 3:**
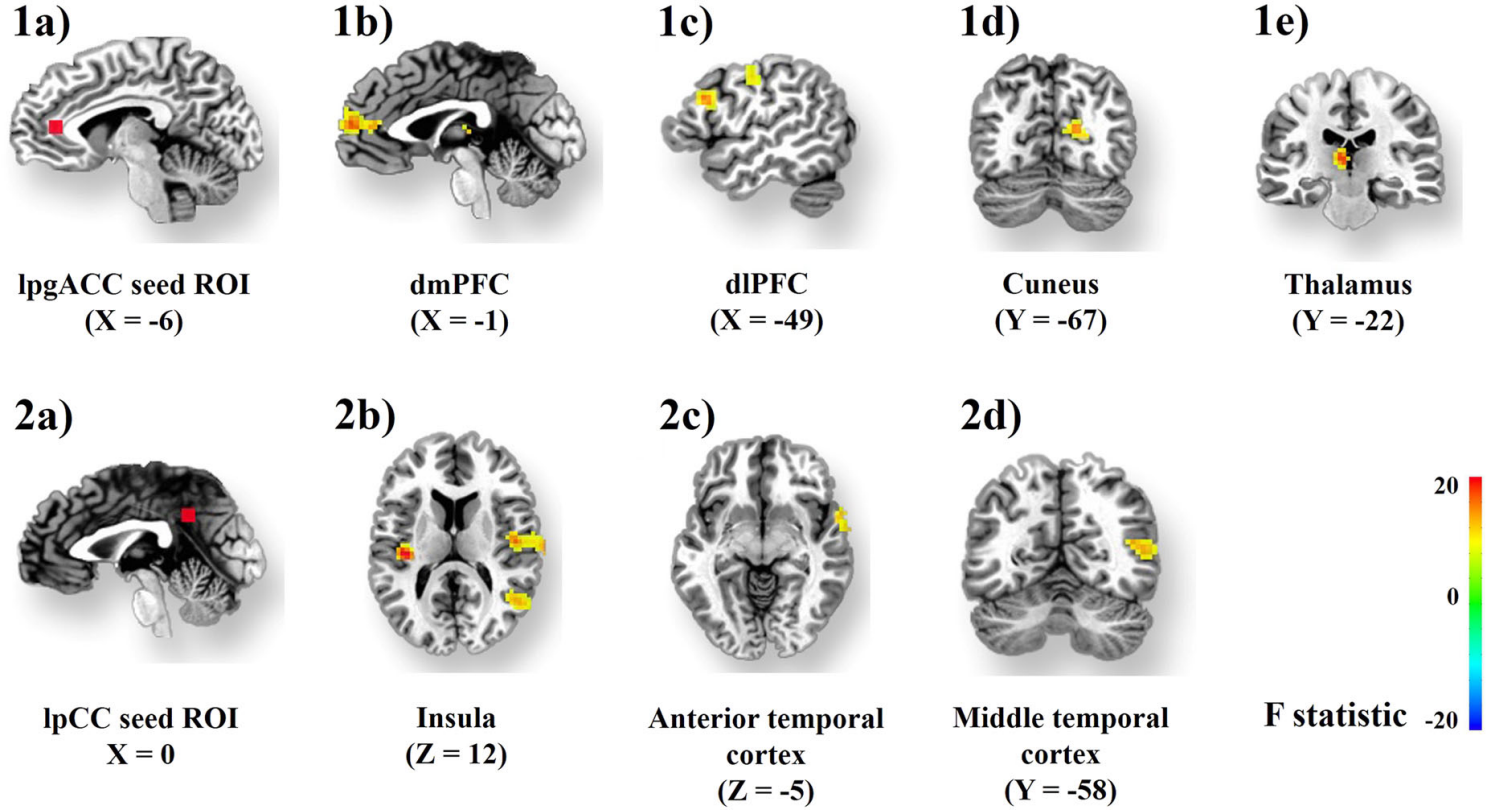
Sex is associated with the iFC of the DMN (default-mode network), the central-executive network (central-executive network), and the salience network (salience network) Upper panel: boys exhibited higher iFC than girls between the lpgACC seed ROI (1**a**) and clusters within the dmPFC (1**b**), dlPFC (1**c**), cuneus (1**d**), and thalamus (1**e**). Bottom panel: boys exhibited higher iFC than girls between the lpCC seed ROI (2**a**), the bilateral insula (2**b**), regions of the anterior (2**c**), and middle temporal cortex (2**d**)

The iFCs of both the lPCC and lpgACC seeds were modulated by sex, but in quite distinct brain regions (Table 5, Fig. 3). Both seeds showed within-DMN iFC clusters. The within-DMN clusters were found in the right lateral temporal cortex (BA38, BA39) for the lPCC seed, and in the right PCC for the lpgACC seed. Both seeds also showed between-network clusters. For the lPCC, these between-network clusters were located in the right and left insula, which are key nodes of the SN. Regarding the lpgACC, the between-network clusters were found in the left mPFC (BA 10) and left dlPFC (BA9), which are key nodes of the central-executive network. In addition, sex modulated an lpgACC-thalamus (pulvinar) cluster.

Finally, the third seed, the lmPFC-iFC revealed a main effect of sex in one cluster located within the right occipital cortex (BA 19) (Table 5).

### Puberty main effect

Other than a main effect of puberty on lmPFC-iFC with the cerebellum (*x* = −13.5, *y* = −79.5, *z* = −33.5; *F* = 20.32; 46 voxels) no clusters were found in cortical or subcortical regions.

### Post-hoc analyses

Based on previous literature, which suggests that small differences in motion during resting-state scans can impact between-group differences in measures of resting-state connectivity^91^, we included average motion per functional volume as a covariate in our analysis of covariance (ANCOVA) models. Controlling for average motion per functional volume did not meaningfully impact the pattern of results reported above (supplemental material, Table S1).

Girls had a significantly higher puberty mean score than boys (Table 1), and *post hoc* analyses demonstrated that puberty-X-sex interactions were characterized by a positive slope in boys and negative slope in girls, as described above. To ensure that interactions between puberty and sex were not driven by a curvilinear (inverted U) relationship, where early pubertal maturation was accompanied by increased iFC (more representative of boys) and late pubertal maturation by decreased iFC (more representative of girls), analyses were repeated while controlling for quadratic effects. These analyses relied on power polynomial regression (supplemental material, Table S2). Puberty-X-sex interactions remained significant after controlling for quadratic effects of puberty.

Another way to control for pubertal differences between girls and boys was to match pubertal maturation between sex groups. Boys exhibited lower pubertal development scores than girls (Table 1), and, reciprocally, low levels of pubertal development ( < 2) were not observed in girls. To ensure that the differential effect of puberty between boys and girls was not driven by non-overlapping developmental stages, we tested for puberty-X-sex interactions within a sample of participants who had pubertal development scores of 2 or greater (supplemental material, Table S3). Importantly, all clusters identified as a function of the interaction within our whole brain analyses remained significant after excluding participants with low PDS scores.

### Exploratory brain–behavior analyses

Multiple regression analyses were conducted to determine whether the three iFC principal components uniquely predicted variance within the three behavioral principal components. The ACC-sex principal component was negatively associated with the principal component characterizing internalizing problems (*β* = −0.139, *p* = 0.028), whereas controlling for other iFC principal components.

## Discussion

To our knowledge, this is the first resting-state fMRI study to examine the effects of puberty and sex, without the confounding effects of chronological age. This study is specifically focused on the resting-state networks that have been previously implicated in youth vulnerability to internalizing problems. The main tenet that drives the present work is that adolescence is a period of huge transformations, particularly at the brain level, and that these changes contribute to the development of psychopathology, such as internalizing disorders. Furthermore, a critical determinant of these changes rests on puberty-related action of sex steroids. The uniqueness of this study is to examine developmental changes within functional brain organization that can be attributed to pubertal maturation, age being held constant, and that these changes are likely to play a role in the vulnerability to internalizing disorders.

Accordingly, the three seeds examined in the present study belong to the DMN^80^. These seeds have also been shown to be associated with subthreshold elevated^40^, as well as depressed^41^ mood symptoms in two independent subsets of a community cohort of 14 yo (IMAGEN consortium^39^). Of note, these cortical regions have also been implicated in the processing of salience and emotion encoding, particularly in the context of social and self-referential information (mPFC)^82^, visual stimuli (PCC)^83^, and autonomic visceral signals (pgACC)^84^.

Based on the Triple-Network Model^55^ and the focus on vulnerability to emotion dysregulation, three predictions regarding the iFC of the DMN were tested. First, pubertal maturation would impact the DMN iFC differently in girls and boys. Second, sex differences in the DMN iFC would be detected independently of puberty. Third, no sex-independent effects of puberty would be found, given that puberty is by essence sexually dimorphic. Finally, brain–behavior relationships would inform the potential contribution of the DMN to the onset of internalizing problems 2 years later. Findings were broadly in line with predictions.

### Puberty-X-sex interaction

As expected, within- and between-network couplings of the DMN were affected by pubertal maturation differently in girls and boys. These effects were restricted to the lmPFC and lPCC seeds (Table 5). The lmPFC and lPCC connectivity maps revealed similar patterns with regards to iFC topography and sex effects. First, both maps revealed within-network and between-network iFC clusters, the latter specifically with the central-executive network, but not the SN. Second, all findings followed the same motif, i.e., connectivity decreased with puberty in girls, but increased in boys. We speculated that this pattern could be relevant to the emergence of affective dysregulation in adolescence, which affects more girls than boys. Accordingly, the observed sexually dimorphic maturational changes of the DMN might reflect changes in adaptive emotion regulation with puberty. The direction of these changes might be protective in boys, or reflect vulnerability in girls for mood dysregulation.

In support of this thesis, the exploratory brain–behavior analysis across the whole sample revealed a negative association between the spgACC iFC component and internalizing symptoms 2 years later: The lower the connectivity at 14 yo, the more severe were internalizing problems at 16 yo. This relationship suggests that puberty, which is accompanied by decreased iFC in girls, might amplify risk for internalizing symptoms in girls. Findings from the literature using clinical samples or individuals at risk for internalizing disorders could inform this interpretation. Unfortunately, results are inconsistent, in terms of topology, direction of group differences, methodology, and sample characteristics. A number of studies in pediatric samples have reported that internalizing symptoms were associated with lower iFC in core networks. For example, Frost-Bellgowan et al. (2015)^92^ reported lower DMN-iFC (with insula, ventral striatum, pre/post central gyrus) in behavioral inhibited (at risk for internalizing disorders) vs. healthy children (8–17 yo), and showed that girls (not boys) exhibited a negative correlation of internalizing-symptom severity with DMN-iFC. Another study of 8–12-yo children also showed that patients with depression/anxiety (vs. healthy peers) had similarly lower iFC, but this time in the ventral attention network, which was the exclusive focus of this work^66^. In contrast, a relatively large study in a community sample of 112 children (53 boys, 59 girls; mean age = 11.5 yo)^93^ focused on the three networks of the triple-network model^55^ in relation to internalizing symptoms (i.e., anxiety and depression, and rumination). Findings revealed no sex differences in the iFCs of the DMN, SN, or CEN. However, brain–behavior correlations identified a positive correlation between iFC within the SN and the levels of internalizing symptoms in girls, but not in boys. Specifically, higher SN-iFC predicted more severe symptoms in girls. In summary, this study^93^ reported a differential effect of sex on the association of SN-iFC with internalizing symptoms, affecting only girls. Compared with our current study, this brain–behavior relationship^93^ concerned a different network (SN) and was in the opposite direction to our findings (greater iFC with higher symptoms, while we showed lower iFC with higher symptoms). Furthermore, it is difficult to compare these results with the present findings, because of the differences in methodology (independent component analysis), and of the significantly younger sample (11yo). Collectively, these pediatric studies concur on the association between dysfunction and internalizing symptoms, but the direction of this dysfunction is inconsistent, as is the specific couplings. In addition, the two studies probing the effect of sex revealed that brain–behavior associations concerned mainly girls. This observation supports our interpretation that the puberty-related reduction in the DMN iFC in girls might carry vulnerability for internalizing symptoms.

Negative findings regarding the distinct iFC modulation by puberty in girls and boys are notable. The lack of sex modulation of the effects of puberty on the lpgACC seed connectivity, and the lack of implication of the SN (particularly the insula) and the amygdala were surprising. Indeed, their established role in the coding of salience and emotional responses (e.g., ref. ^94,95^, would be expected to contribute to the increased emotional intensity and lability in adolescence (e.g., ref. ^60,64,96^). These negative findings suggest that the iFC of the nodes/circuits underlying emotion/motivation processes do mature similarly in boys and girls. Although task-based fMRI studies have reported puberty-related changes of these structures in response to emotion/motivation probes (e.g., ref. ^20,22,24^), how sex influences these trajectories has not been reported.

### Sex main effect

In fact, the present findings reveal sex effects, independent of puberty, in the spgACC iFC and SN (insula). In all cases, connectivity was higher in boys than girls. These sex effects concerned mainly two of the three seeds, the lPCC and lpgACC.

From the perspective of the Triple-Network Model, sex impacted differently the between-network connectivity of the anterior (spgACC seed) and posterior (lPCC seed) DMN components (Table 5) (anterior vs. posterior hubs of the DMN^97^). Sex influenced the connectivity of the anterior DMN seed (spgACC) with the SN, and the connectivity of the posterior seed (lPCC) with the central-executive network. In line with this distinction, as discussed above, the anterior spgACC connectivity predicted internalizing symptoms 2 years later (age 16 years), whereas the posterior lPCC component was not associated with any of the behavioral components.

These differential effects of sex on the anterior and posterior DMN iFC need to be examined more closely in the context of the modulation of the Triple-Network Model, and might suggest a refinement of this model by considering regional functional specialization within the DMN, i.e., the anterior and posterior aspects. Accordingly, they suggest that the modulation by sex and puberty affects differently the anterior and posterior DMN components^98^, which likely play distinct roles in vulnerability to emotion dysregulation.

### Limitations

These findings are not without limitations. First, puberty was measured by self-report (PDS^71^), without physical examination or hormonal assays. However, the PDS has been shown to have good psychometric properties and convergent validity from self- and physician-rated Tanner stages^71,72^. Second, as girls and boys were of similar age, the average pubertal level was higher in girls. Two different control analyses were conducted to validate the findings. First, the youngest boys were removed from analysis to equalize the puberty mean in girls and boys. Findings with these control analyses revealed that the pattern of results remained pretty much unchanged (supplemental material, Table S3). Second, we conducted multiple regression analyses, which included quadratic terms for puberty across the whole sample. This approach explored whether the effects of puberty were indeed characterized by two separate and opposite lines of regression in girls and boys, while controlling for an inverse-U-curve pattern of pubertal maturation. Results showed that the puberty-X-sex interaction was still significant (supplemental material, Table S2). Therefore, we are confident in the findings of different pubertal trajectories in boys and girls. Third, this study was cross-sectional, which is sub-optimal for the study of developmental changes. However, this limitation is leveraged by the relatively large sample size, and also the homogeneous age that allowed us to dissociate the effects of chronological changes from those specific to puberty. Fourth, this study focused on extending earlier brain structural findings of neural risk for mood problems in 14 yo. Accordingly, only three seeds were investigated, which constrained the yield of this study. However, this approach is specific to a behavioral domain that, we hope, will foster the formulation of models of vulnerability to internalizing problems to be tested in the future.

## Conclusions

This study reveals that pubertal maturation influences iFC differently in boys and girls, and that sex can impact iFC independently of puberty. First, because mood dysregulation has its peak onset in adolescence, during pubertal maturation, it is reasonable to consider a role of puberty in the rise of incidence rate of mood symptoms. Second, because mood dysregulation, particularly depression and anxiety, occurs disproportionally in girls, sex is expected to uniquely modulate circuits of emotion regulation. For these reasons, analyses were focused on three regions previously identified as conferring risk for mood problems in adolescents. These three regions happened to belong to the DMN, which is recognized to be perturbed in pathological mood disturbances. Findings revealed that both puberty-X-sex interaction and sex main effects modulate within-network and between-network clusters of the DMN iFC. Notably, effects of puberty did not involve the amygdala or the SN, suggesting the notion that pubertal maturation might not significantly affect the iFC of these key centers of emotion processes. This is in contrast to the effect of sex, which did impact DMN–SN iFC. Tentatively, stronger iFC in boys might suggest tighter emotional control, also potentially serving a protective role against emotion dysregulation. Finally, the spgACC iFC significantly predicted internalizing symptoms 2 years later, supporting the association of this network with mood dysregulation.

## Supplementary information

Suppl. Material
