# Supplementary material for: Pubertal maturation and sex effects on the default-mode network connectivity implicated in mood dysregulation
Source: Transl Psychiatry. 2019 Feb 25;9:103. doi: 10.1038/s41398-019-0433-6 (PMC6389927; doi:10.1038/s41398-019-0433-6)
Supplement: Supplementary file 1 — Suppl. Material [file 41398_2019_433_MOESM1_ESM.docx]

**Supplemental Materials**

**Supplemental Methods**

## *BOLD fMRI Data Acquisition*

MRI data were acquired at five sites using 3T scanners: Phillips (Dublin), General Electrics (London), and Siemens (Paris, Dresden, Mannheim). BOLD fMRI signal was acquired across 40 interleaved slices using the following parameters: TR = 2,200 ms; TE = 30 ms; flip angle = 75°; acquisition matrix = 64 × 64 × 40 with 2.4 mm slice thickness and 1 mm slice gap yielding an acquisition resolution 3.4 mm isotropic; 187 volumes collected over 6.5 minutes. High resolution anatomical images were obtained using parameters based on the ADNI protocol, yielding a final voxel size of 1.1 x 1.1 x 1.1 mm.

*Regions of Interest*

Seed regions-of-interest (ROIs) were created based on coordinates from previous analyses reporting reductions in gray matter volume associated with subthreshold bipolar symptoms (31). MNI coordinates were all converted to Talairach coordinates using the Yale mni2tal GUI (“MNI- Yale University” 2017). Specifically, cubic ROIs (3mm x 3mm x 3mm) were created within the left pregenual ACC (lpgACC; Talairach: x = -12, y = 36, z = 12), left medial PFC (lmPFC; Talairach: x = -2, y = 45, z = 16), and the left PCC (Talairach: x = -1, y = -47, z = 34 (58).

**Supplemental Results:**

Based on previous literature which suggests that small differences in motion during resting state scans can impact between group differences in measures of resting state connectivity (Van Dijk, Sabuncu, & Buckner, 2012), we tested whether participant motion during the resting state scan varied as a function of our variables of interest.

Age (R = -0.14, p < 0.02) and puberty (R = -0.15, p < 0.01) were both associated with average motion per functional volume, such that younger participants and participants at lower levels of pubertal development exhibited more motion during the resting state scan. Average motion per functional volume also differed between imaging centers (F = 7.29, p < 0.001). Boys had higher levels of motion than girls, but this difference was not statistically significant (T = 1.49, p = 0.14).

To ensure that between subject differences in motion did not contribute to the significant of our results, average motion per functional volume was added as a covariate to our ANCOVA models testing for effects of puberty, sex, and puberty by sex interactions. Controlling for average motion per functional volume, did not meaningfully impact the pattern of results reported (for full details, see Supplemental Table S1).

**Supplemental Tables:**

**Table S1**. Results including group motion correction (ANCOVA: average motion per TR). Italics: cluster size< 37. Results reported in Talairach coordinates.

|  |  |  |  |  | **Talaraich** | | | |
| --- | --- | --- | --- | --- | --- | --- | --- | --- |
| **Seed** | **Analysis** | **Region** | **Cluster Size (k)** | **Maxima** | **X** | **Y** | **Z** |  |
| **L mPFC** | **PxS** | dmPFC (BA 6) | 159 | 16.14 | -4.5 | 4.5 | 56.5 |  |
|  |  | *R mTemporal Ctx* | *32* | *15.11* | *58.5* | *-55.5* | *-6.5* |  |
|  |  | *R dlPFC (BA 9)* | *28* | *10.81* | *40.5* | *34.5* | *29.5* |  |
|  | **Sex** | R Occipital Ctx | 38 | 18.44 | 31.5 | -88.5 | 17.5 |  |
|  | **Puberty** | none |  |  |  |  |  |  |
| **L PCC** | **PxS** | L Inferior Parietal Ctx | 74 | 23.23 | -43.5 | -46.5 | 41.5 |  |
|  |  | L Precentral Ctx (BA 6) | 50 | 14.22 | -46.5 | 1.5 | 32.5 |  |
|  |  | R Precentral Ctx (BA 6) | 43 | 11.24 | 46.5 | 1.5 | 26.5 |  |
|  |  | *dmPFC (BA 8)* | *36* | *15.23* | *7.5* | *25.5* | *44.5* |  |
|  |  | R mTemporal Ctx | 55 | 15.45 | 58.5 | -52.5 | -9.5 |  |
|  | **Sex** | L Insula | 43 | 22.49 | -37.5 | -22.5 | 11.5 |  |
|  |  | R Insula | 91 | 16.59 | 46.5 | -13.5 | 11.5 |  |
|  |  | R mTemporal Ctx | 56 | 13.40 | 49.5 | -58.5 | 14.5 |  |
|  |  | R aTemporal Ctx | 47 | 16.84 | 58.5 | 7.5 | -6.5 |  |
|  | **Puberty** | Cerebellum | 38 | 19.96 | -13.5 | -79.5 | -36.5 |  |
| **L ACC** | **PxS** | none |  |  |  |  |  |  |
|  | **Sex** | L mPFC (BA 10) | 142 | 16.53 | -1.5 | 55.5 | 14.5 |  |
|  |  | L dlPFC (BA 9) | 109 | 15.88 | -49.5 | 16.5 | 26.5 |  |
|  |  | *R PCC* | *36* | *15.46* | *13.5* | *-67.5* | *14.5* |  |
|  |  | L Thalamus | 48 | 17.72 | -7.5 | -22.5 | 8.5 |  |
|  | **Puberty** | none |  |  |  |  |  |  |

**Table S2**. Skew, Kurtosis, and Levene's test for equality of variances performed on extracted values from clusters exhibiting main effects of puberty, sex, and their interaction. F statistics and p values reflect the equality of variances between high and low puberty scores determined by mean split (left column), and between boys and girls (right column).

|  | **Puberty** | | | **Sex** | | **Skew** | **Kurtosis** |
| --- | --- | --- | --- | --- | --- | --- | --- |
|  | **Region** | **F statistic** | **p value** | **F statistic** | **p value** |  |  |
| **Puberty X Sex Interaction** | | | | | | | |
| **lMPFC-seed** | L dmPFC (BA 6) | 1.20 | 0.28 | 0.94 | 0.33 | 0.53 | 0.26 |
|  | R dlPFC (BA 9) | 0.61 | 0.44 | 0.004 | 0.95 | 0.42 | 0.45 |
|  | R mTemporal ctx | 0.07 | 0.80 | **4.76** | **0.03** | 0.22 | 0.55 |
| **lPCC-seed** | R dmPFC (BA 8) | 0.10 | 0.76 | 0.02 | 0.90 | 0.31 | 0.66 |
|  | L Precentral Ctx (BA 6) | 1.14 | 0.29 | 1.95 | 0.16 | 0.42 | 0.60 |
|  | R Precentral Ctx (BA 6) | 1.06 | 0.30 | 3.74 | 0.054 | 0.32 | 0.68 |
|  | R mTemporal Ctx | 0.60 | 0.44 | 2.00 | 0.16 | 0.45 | 0.80 |
|  | L Inferior Parietal Ctx | 0.02 | 0.89 | 0.02 | 0.90 | 0.13 | 0.04 |
| **Main Effect of Sex** | | | | | | | |
| **lMPFC-seed** | R Occipital Cx | 3.74 | 0.054 | 0.00003 | 0.996 | 0.58 | 1.69 |
| **lPCC-seed** | L Insula | 1.14 | 0.29 | 3.71 | 0.055 | 0.69 | 0.72 |
|  | R Insula | 0.02 | 0.89 | 3.03 | 0.083 | 0.58 | 0.06 |
|  | R mTemporal Ctx | 0.003 | 0.95 | 0.12 | 0.73 | 0.12 | -0.34 |
|  | R aTemporal Ctx | 0.19 | 0.66 | 0.38 | 0.54 | 0.54 | 0.40 |
| **lpgACC-seed** | L mPFC (BA 10) | 2.72 | 0.10 | 2.63 | 0.11 | 0.61 | 1.28 |
|  | L dlPFC (BA 9) | **8.58** | **0.004** | **15.19** | **< .001** | **1.05** | **2.26** |
|  | R PCC | **6.35** | **0.012** | **15.10** | **< .001** | 0.75 | 0.98 |
|  | L Thalamus | 0.00008 | 0.99 | **4.89** | **0.03** | 0.19 | 0.36 |
| **Main Effect of Puberty** | | | | | | | |
| **lMPFC-seed** | Cerebellum | 1.12 | 0.29 | 0.63 | 0.43 | 0.21 | 0.08 |

**Table S3: Multiple regression models.** Statistics associated with the interaction of puberty and sex predicting iFC values from clusters of interest (middle column), including beta coefficients and p values from multiple regression models which statistically control for curvilinear (quadratic) effects of puberty. All models presented in this table control for age and scanner site.

|  | **Models without puberty power polynomial** | | **Models with puberty power polynomial** | |
| --- | --- | --- | --- | --- |
| **mPFC** |  |  |  |  |
|  | **ß value** | **P value** | **ß value** | **P value** |
| dmPFC | -.334 | < .001 | -.316 | =.003 |
| Middle temporal gyrus | -.307 | < .001 | -.375 | =.001 |
| Dorsolateral PFC | -.305 | < .001 | -.275 | =.013 |
| **PCC** |  |  |  |  |
|  | **ß value** | **P value** | **ß value** | **P value** |
| Inferior parietal lobule | -.342 | < .001 | -.410 | < .001 |
| Left precentral gyrus | -.319 | < .001 | -.375 | =.001 |
| Right Precentral gyrus | -.302 | < .001 | -.324 | =.003 |
| dmPFC | -.298 | < .001 | -.339 | =.002 |
| Middle temporal gyrus | -.318 | < .001 | -.384 | <.001 |

**Table S4: Puberty stage** >**2.** Statistics associated with the interaction of puberty and sex predicting iFC values from clusters of interest (middle column), including beta coefficients and p values from multiple regression models excluding participants with a pubertal development score (PDS) less than 2. All models presented in this table control for age and scanner site.

|  | **Full Sample (N = 304)** | | **Participants with PDS > 2 (N = 270)** | |
| --- | --- | --- | --- | --- |
| **mPFC** |  |  |  |  |
|  | **ß value** | **P value** | **ß value** | **P value** |
| dmPFC | -.334 | < .001 | -.234 | =.030 |
| Middle temporal gyrus | -.307 | < .001 | -.381 | =.001 |
| Dorsolateral PFC | -.305 | < .001 | -.265 | =.017 |
| **PCC** |  |  |  |  |
|  | **ß value** | **P value** | **ß value** | **P value** |
| Inferior parietal lobule | -.342 | < .001 | -.361 | = .001 |
| Left precentral gyrus | -.319 | < .001 | -.321 | =.003 |
| Right Precentral gyrus | -.302 | < .001 | -.323 | =.003 |
| dmPFC | -.298 | < .001 | -.240 | =.025 |
| Middle temporal gyrus | -.318 | < .001 | -.397 | <.001 |

**Table S5:** Decomposition of the Puberty by Sex significant effects on iFC. Correlations between puberty and significant iFC clusters are shown separately for boys and girls, for the lmPFC seed and the lPCC seed.

| **Effect of Puberty on iFC** | **Boys** | | **Girls** | |
| --- | --- | --- | --- | --- |
|  |  |  |  |  |
| **lmPFC-seed** |  |  |  |  |
|  | **ß value** | **P value** | **ß value** | **P value** |
| dmPFC | 0.080 | < 0.0005 | -0.073 | < 0.05 |
| Middle temporal gyrus | 0.062 | < 0.005 | - 0.084 | < 0.01 |
| Dorsolateral PFC | 0.038 | = .071 | -0.100 | < 0.005 |
| **lPCC-seed** |  |  |  |  |
|  | **ß value** | **P value** | **ß value** | **P value** |
| Inferior parietal lobule | 0.074 | < 0.005 | -0.104 | < 0.005 |
| Left precentral gyrus | 0.046 | < 0.05 | - 0.108 | < 0.001 |
| Right Precentral gyrus | 0.040 | = 0.089 | - 0.114 | < 0.001 |
| dmPFC | 0.075 | < 0.005 | -0.078 | < 0.05 |
| Middle temporal gyrus | 0.073 | < 0.005 | - 0.103 | <0.01 |
|  |  |  |  |  |

Beta coefficients and p values associated with the simple slopes between pubertal development and iFC are presented separately for boys (middle column) and girls (right column). All models presented in this table control for age and scanner site.

Labels: mPFC: medial prefrontal cortex; dmPFC: dorsomedial prefrontal cortex; PCC: posterior cingulate cortex
